# Associations Between Maternal Pre-Pregnancy Dietary Patterns and Body Composition in Preschool Children: A Prospective Cohort Study

**DOI:** 10.3390/nu18172895

**Published:** 2026-09-03

**Authors:** Qi Zhang, Yongli Zhao, Yifan Duan, Guina Luo, Jingtao Duan, Changqing Liu

**Affiliations:** 1 Institute for Nutrition and Food Safety, Hebei Provincial Center for Disease Control and Prevention, Shijiazhuang 050021, China; zhangqi10162024@126.com (Q.Z.);; 2 National Institute for Nutrition and Health, Chinese Center for Disease Control and Prevention, Beijing 100050, China; 3 Department of Epidemiology, Wuqiang Center for Disease Control and Prevention, Hengshui 053000, China

**Keywords:** pre-pregnancy dietary patterns, body composition, preschool children, prospective cohort study, principal component analysis

## Abstract

Background: Maternal nutritional status before pregnancy may influence offspring long-term health; however, the association between pre-pregnancy dietary patterns and body composition in preschool children remains unclear. Methods: This study used data from the Taicang and Wuqiang Mother–Child Cohort Study (TAWS), comprising 582 mother–child pairs. Dietary intake during the month before pregnancy was assessed using a food frequency questionnaire. Dietary patterns were derived via principal component analysis. Standardized pattern scores (mean = 0, SD = 1) were analyzed as continuous variables (per 1-SD increase) and categorized into quartiles (Q1–Q4, with Q1 as reference). Body composition indicators (percent body fat [PBF], fat mass index [FMI], fat-free mass index [FFMI], and skeletal muscle index [SMI]) were measured by bioelectrical impedance analysis in children aged 3–6 years. Multiple linear regression was used to examine associations under two models: an unadjusted model and a multivariable model adjusted for child characteristics, maternal sociodemographic and gestational factors, family factors, feeding mode, and other dietary patterns. Interaction terms were introduced to test for potential effect modification by child sex and maternal pre-pregnancy BMI. Results: Results showed that five maternal pre-pregnancy dietary patterns were extracted: animal-based food, grain-vegetable-fruit, processed meat-dairy, vegetable-legume, and cured vegetable. In multivariable-adjusted models, the processed meat-dairy pattern was positively associated with FMI (Q4 vs. Q1: β = 0.262, 95% CI: 0.036 to 0.488). The grain-vegetable-fruit pattern was inversely associated with FFMI (Q4: β = −0.265, 95% CI: −0.510 to −0.020) and SMI (Q4: β = −0.256, 95% CI: −0.564 to −0.113). The vegetable-legume pattern was associated with higher PBF (Q3: β = 0.234, 95% CI: 0.007 to 0.461) and lower SMI (Q4: β = −0.338, 95% CI: −0.564 to −0.113). No significant associations were observed for the animal-based food or cured vegetable pattern after adjustment. Sex-stratified analyses showed that the animal-based food pattern was associated with FFMI and SMI in opposite directions between boys and girls (*p* for interaction = 0.048 and 0.013, respectively). Maternal pre-pregnancy BMI significantly modified only the association between the processed meat–dairy pattern and SMI (*p* for interaction = 0.032). Conclusions: Maternal pre-pregnancy dietary patterns were selectively associated with body composition in preschool children: the processed meat–dairy pattern was associated with higher fat mass, while the grain-vegetable-fruit and vegetable-legume patterns were associated with lower muscle indices. These associations are observational in nature and do not imply causality. In particular, the associations of plant-based dietary patterns with muscle indices should be interpreted with caution and should not be regarded as negative effects of plant-based diets.

## 1. Introduction

Childhood overweight and obesity have emerged as major global public health concerns. According to a 2022 report from the World Health Organization (WHO), more than 390 million children and adolescents aged 5–19 years worldwide are overweight, including more than 160 million who are obese [1]. The Lancet Global Burden of Disease Study further projects that, by 2050, the prevalence of obesity among children aged 5–14 years will reach 15.6%, affecting approximately 186 million individuals [2]. The prevalence of overweight and obesity among preschool children in China is 18.4% and 10.3%, respectively [3]. Early abnormalities in childhood body composition often precede clinical obesity and may have lasting adverse effects on long-term cardiometabolic health [4].

The Developmental Origins of Health and Disease (DOHaD) theory posits that environmental exposures during critical early developmental stages—including the preconception period—can exert persistent effects on subsequent phenotypes and disease susceptibility through epigenetic modifications and metabolic programming [5]. Increasing evidence indicates that maternal nutritional status prior to conception serves as a significant independent predictor of offspring health outcomes, independent of prenatal exposures [6]. For instance, preconception intake of micronutrients (e.g., folic acid and iron) is closely associated with placental weight, angiogenesis, and transport function, thereby influencing fetal nutrient supply [7]; preconception dietary patterns have also been demonstrated to significantly correlate with fetal growth trajectories, with the Mediterranean diet being linked to reduced risks of small-for-gestational-age infants, whereas Western dietary patterns may increase risks of large-for-gestational-age infants [8]. Furthermore, the long-term associations between maternal body mass index (BMI) and dietary quality before conception and childhood obesity, adipose tissue accumulation, and metabolic abnormalities have been validated in multiple prospective cohorts [9]. However, existing studies predominantly focus on prenatal diets or single-nutrient supplementation, leaving scarce direct evidence linking preconception dietary patterns to body composition—particularly adipose tissue and muscle indicators—in preschool children. Traditional approaches overemphasize individual nutrients while often overlooking the complex integrated nature of food intake [10]. In contrast, compared with analyses of single nutrients or specific foods, dietary pattern analysis comprehensively reflects overall dietary quality and better captures the genuine relationship between diet and health [11]. Therefore, this study employs dietary pattern analysis to systematically investigate the relationship between preconception diet and body composition in preschool children.

In recent years, the associations between pre-pregnancy and pregnancy dietary intake and offspring body composition have received increasing attention; however, direct prospective evidence focusing on the pre-conception period remains limited and inconsistent. The Southampton Women’s Survey found that poorer maternal pre-pregnancy diet quality was associated with increased body fat percentage and decreased lean body mass in offspring at 8–9 years of age [9]. The Australian Longitudinal Study on Women’s Health also indicated that higher pre-pregnancy diet quality was associated with reduced risk of offspring obesity and low birth weight [12]. In contrast, more robust evidence is available regarding the associations of gestational diet with offspring body composition. The Nurses’ Health Study II observed a trend toward a positive association between higher gestational intake of processed food and offspring overweight, although this did not reach statistical significance [13]. A meta-analysis of seven European birth cohorts demonstrated that higher gestational diet quality reduced the risk of childhood overweight and obesity [14]. Greater dietary diversity during pregnancy was associated with favorable neonatal body composition [15]. Adherence to a “vegetables-fish-fat” dietary pattern in early pregnancy was associated with lower BMI and body fat percentage in children at age 6 years [16]. Conversely, higher gestational protein intake may increase the risk of overweight and obesity in children at 7 and 11 years of age [17]. These associations may be mediated by mechanisms such as chronic low-grade inflammation [18], disturbances in insulin and glucose homeostasis [19], and epigenetic modifications in genes involved in energy balance and lipogenesis [20].

However, most of the aforementioned studies have focused on dietary patterns during pregnancy, and prospective evidence directly linking pre-pregnancy dietary habits to body composition—particularly fat and muscle indicators—in preschool children remains scarce. Moreover, current research often pools data from both sexes, overlooking the sex-specific programming effects inherent in the DOHaD framework. Animal model studies consistently demonstrate that a maternal high-fat diet induces obesity more strongly in male than in female offspring [21]; additionally, significant sex differences exist in the association between maternal dietary quality and offspring metabolic parameters. Thus, simply merging data from both sexes may obscure genuine biological correlations [22]. Furthermore, the impact of maternal diet on fetal intrauterine development may be substantially influenced by pre-pregnancy body mass index (BMI), as both pre-pregnancy overweight and obesity independently affect appetite regulatory function in offspring.

Although a limited number of prospective studies have examined the association between pre-pregnancy diet and offspring body composition [9,12], few have simultaneously investigated the relationship between pre-pregnancy dietary patterns and multiple body composition parameters, including adipose tissue and muscle indicators, in preschool children, or systematically analyzed the moderating effects of offspring sex and maternal pre-pregnancy BMI. Therefore, this study aims to (i) identify maternal pre-pregnancy dietary patterns and analyze their associations with preschool children’s body composition parameters (percent body fat [PBF], fat mass index [FMI], fat-free mass index [FFMI], and skeletal muscle index [SMI]); (ii) conduct stratified analyses by offspring sex and maternal pre-pregnancy BMI (<24 kg/m^2^ and ≥24 kg/m^2^) to assess sex-specific associations and BMI’s moderating effects [23,24]; (iii) evaluate dose–response relationships through trend analysis.

The study tests the following hypotheses based on the literature reviewed above and DOHaD theory: (1) Maternal pre-pregnancy dietary patterns are significantly associated with body composition indicators in preschool children; (2) Animal-based food and processed meat-dairy diets are positively associated with elevated fat indices (PBF, FMI), whereas the associations of plant-based dietary patterns (grains-fruits-vegetables, vegetables-legumes) with muscle indices (FFMI, SMI) are examined as exploratory analyses without a strong a priori directional hypothesis; (3) These associations differ by child gender and are modified by maternal pre-pregnancy BMI.

## 2. Materials and Methods

### 2.1. Study Design and Population

This study utilized data from the Hebei Wuqiang sub-cohort of the China Maternal and Infant Nutrition and Health Cohort Study, the Taicang and Wuqiang Mother–Child Cohort Study (TAWS), an ongoing Chinese maternal and child nutrition and health cohort. Further details regarding TAWS have been published elsewhere [25,26]. The project was approved by the Ethics Committee of the National Institute for Nutrition and Health, Chinese Center for Disease Control and Prevention (No. 2016-014), and all participants provided written informed consent. Women were enrolled during early pregnancy between June 2016 and December 2018. This study used a prospective cohort design, with body composition of preschool children as the primary outcome. The exposure—maternal dietary patterns in the month before pregnancy—was retrospectively assessed using a Food Frequency Questionnaire (FFQ) that has been previously validated among Chinese women of reproductive age and has demonstrated good reproducibility and reasonable validity for major nutrient intakes [25,26] at enrollment in early pregnancy. Thus, the outcome assessment was prospective, whereas the exposure assessment was retrospective. Maternal pre-pregnancy dietary information and baseline characteristics were collected at enrollment during early pregnancy, and a preschool follow-up was conducted between September and November 2022, during which anthropometric measurements and questionnaires were uniformly completed in kindergartens.

**Figure 1:**
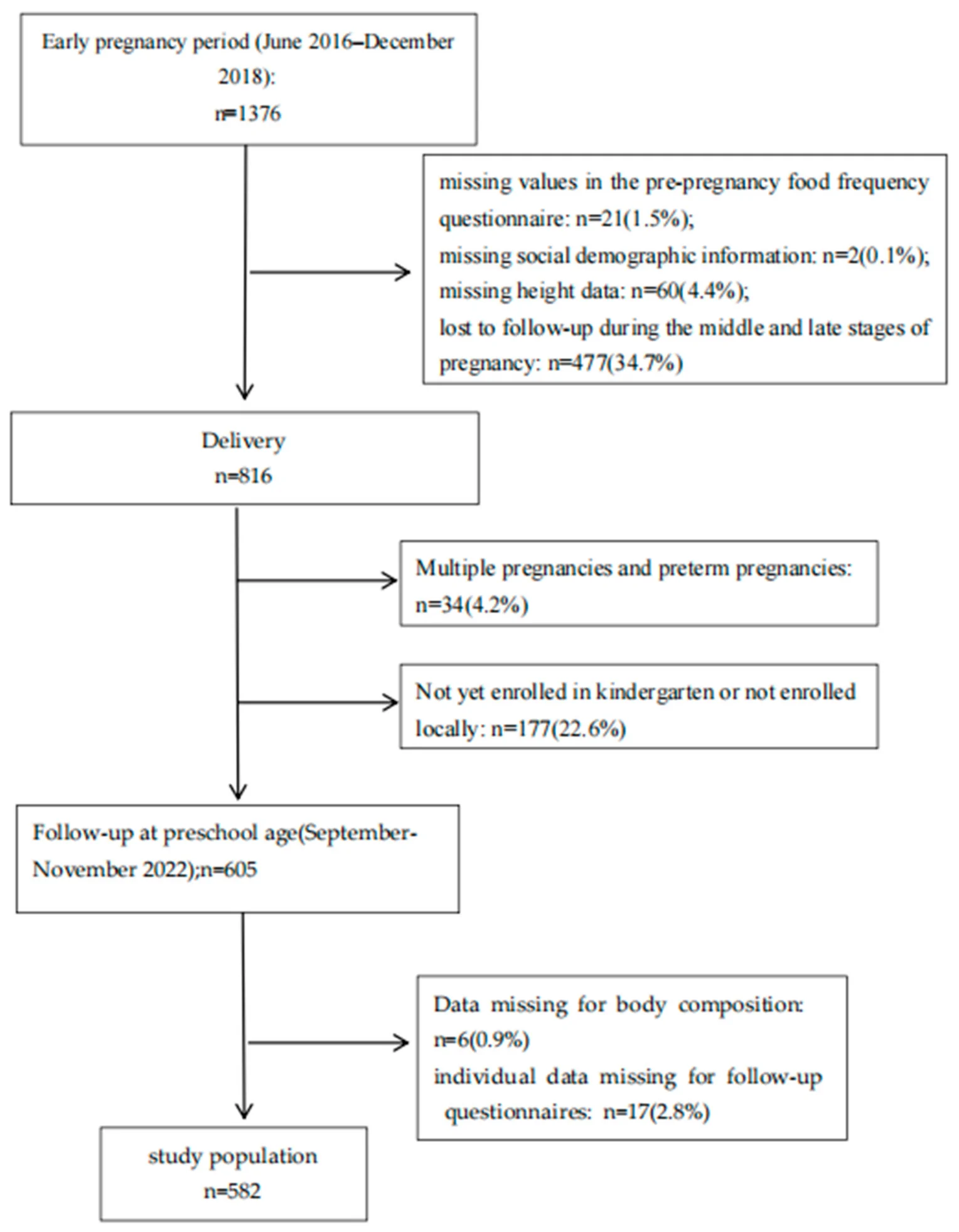
Flow chart of study subject inclusion and exclusion. Note: The initial cohort comprised 1376 women enrolled during early pregnancy (June 2016–December 2018). Of these, 560 women were lost during pregnancy (including 21 with missing pre-pregnancy FFQ data, 2 with missing sociodemographic information, 60 with missing height data, and 477 lost to follow-up during mid-to-late pregnancy), leaving 816 singleton live births. After excluding 34 multiple pregnancies and preterm deliveries, and 177 children not enrolled in kindergarten or not enrolled locally at the time of follow-up (September–November 2022), 605 mother–child pairs completed the preschool follow-up. The follow-up completion rate was calculated as 605/782 × 100% = 77.4%, where 782 represents the number of singleton live births eligible for preschool follow-up (i.e., after excluding multiple pregnancies and preterm deliveries from the 816 deliveries). An additional 23 pairs were excluded due to missing body composition data (*n* = 6) or incomplete follow-up questionnaires (*n* = 17), yielding a final analytic sample of 582. The 177 children not enrolled locally were primarily attributable to family relocation or not having reached the minimum kindergarten enrollment age (3 years) at follow-up. These reasons for loss to follow-up are considered external factors unlikely to be directly associated with maternal pre-pregnancy dietary patterns or baseline health indicators; however, because outcome data are completely missing for these participants, we are unable to statistically test whether the missing mechanism is non-random (MNAR). This limitation is discussed in the manuscript.

The inclusion criteria were: (i) pregnant women aged 18–45 years with gestational age < 16 weeks; (ii) no history of diabetes, hypertension, or major chronic diseases, and in good physical health; (iii) planning to undergo antenatal examinations at the local township health center or Wuqiang County Maternal and Child Health Hospital; (iv) permanent residents of Wuqiang County; and (v) complete anthropometric measurements and questionnaire data for preschool children. A total of 582 preschool children were ultimately included, with the detailed inclusion and exclusion flowchart presented in Figure 1.

### 2.2. Dietary Assessment

Pre-pregnancy dietary intake was assessed at the first visit using a food frequency questionnaire (FFQ). All participants completed the FFQ through one-on-one, face-to-face interviews conducted by trained research staff, with food photographs used as visual aids. For each food item, participants reported both consumption frequency and portion size. Dietary intake was then calculated as grams per day by converting reported frequencies into daily amounts. The 57 food items were grouped into 16 food groups, and daily intake for each group was calculated [27,28,29]. Dietary patterns were extracted using Principal Component Analysis (PCA). Prior to analysis, the Kaiser-Meyer-Olkin (KMO) measure of sampling adequacy and Bartlett’s test of sphericity were computed to assess whether the data were suitable for PCA. Varimax orthogonal rotation was applied to achieve a clearer and more interpretable factor structure. The number of principal components retained was determined by the following three criteria: (1) the Kaiser criterion (i.e., retention of components with initial eigenvalues > 1.0); (2) the inflection point (elbow) of the scree plot; and (3) the nutritional interpretability of each component after rotation. Food groups with absolute factor loadings greater than 0.3 were considered major contributors to a given dietary pattern, informing pattern naming and interpretation. Dietary pattern factor scores were calculated for each participant, with higher scores indicating greater adherence to that pattern. Dietary pattern factor scores were standardized and then categorized into quartiles (Q1–Q4) using the percentile method, with Q1 representing the lowest adherence and Q4 the highest adherence. The intake of core food groups increased progressively across quartiles, indicating that the grouping was nutritionally interpretable.

### 2.3. Measurement of Body Composition in Preschool Children

Height was measured using a metal stadiometer (SG-210, Yuejian Fitness Equipment Co., Ltd., Nantong, China). Body weight and body composition were assessed with a bioelectrical impedance analyzer (InBody 770, Biospace Co., Ltd., Shanghai, China). Body composition parameters included total body fat mass, fat-free mass, total body water, skeletal muscle mass, bone mineral mass, as well as fat and fat-free mass in the trunk and extremities, and visceral fat area.

The primary outcomes in this study were percent body fat (PBF), fat mass index (FMI), fat-free mass index (FFMI), and skeletal muscle index (SMI). Percent body fat was defined as the percentage of total body fat mass relative to total body weight. Because body fat, fat-free mass, and skeletal muscle mass are all correlated with height, height-adjusted indices were calculated: FMI was computed as fat mass (kg) divided by height squared (m^2^); FFMI as fat-free mass (kg) divided by height raised to the power of 2.5 (m^2.5^) [30]; and SMI as skeletal muscle mass (kg) divided by height squared (m^2^).

### 2.4. Covariates

A comprehensive range of maternal and child characteristics were assessed as potential confounders. In early pregnancy, face-to-face interviews were conducted by trained healthcare professionals to collect information on maternal dietary intake during the month before pregnancy, sociodemographic characteristics (maternal age, education level, family monthly per capita income, etc.), pre-pregnancy weight, height, parity, physical activity, last menstrual period, smoking and alcohol consumption in the six months before pregnancy, and exposure to secondhand smoke during pregnancy. Basic information on fathers (height, weight, education level, etc.) was also collected. Delivery information, including birth outcomes (gestational age at delivery, birth weight, etc.), weight at labor admission, as well as use of nutritional supplements during pregnancy and diagnoses of gestational diabetes mellitus and gestational hypertensive disorders during pregnancy, was extracted from hospital medical records.

Data on feeding behaviors at 1, 2, 3, 6, 8, 12, 18, 24, and 36 months of age were collected through electronic questionnaires. The questionnaire design and indicator calculations were based on the WHO Infant and Young Child Feeding Assessment Indicators [31]. The survey captured consumption (yes/no and frequency) during the preceding 24 h for the following items: breast milk, plain water, infant formula, regular formula, cow’s milk, goat’s milk, yogurt, rice soup, sugar water, fruit juice, tea, beverages, and solid and semi-solid foods.

“Exclusive breastfeeding” was defined as receiving only breast milk, or breast milk with only small amounts of plain water or medications, vitamin and mineral supplements, and oral rehydration salts, without any other foods or liquids, in at least three follow-up visits before 6 months of age. “Formula feeding” was defined as the infant having consumed formula milk at any follow-up visit before 6 months of age, without receiving any other liquid milk or solid/semi-solid foods.

At the preschool follow-up, children’s dietary intake during the previous month was evaluated using a food frequency questionnaire (FFQ). In-kindergarten dietary data were obtained from kindergarten menus with assistance from class teachers and cafeteria staff, while out-of-kindergarten dietary data were reported by primary caregivers. Overall dietary intake was calculated by weighting and combining in-kindergarten and out-of-kindergarten data according to attendance. Additional child data—including physical activity, sleep duration, water consumption, nutritional supplement use, illness episodes (diarrhea, fever, pneumonia), and attendance during the past month—were collected via questionnaires. Kindergarten-level characteristics, such as class size, drinking habits, physical activity schedules, and sleep arrangements, were also documented.

### 2.5. Statistical Analysis

Statistical analyses were performed using R version 4.5.1. Continuous variables were described as mean ± standard deviation (x±s), categorical variables as *n* (%), and non-normally distributed continuous variables as median (Q1, Q3). Pre-pregnancy dietary patterns were extracted using principal component analysis (PCA) with varimax orthogonal rotation; factors with eigenvalues >1 and absolute factor loadings >0.3 were retained. Factor scores were calculated for each pattern, with higher scores indicating greater adherence. Standardized dietary pattern factor scores (mean = 0, SD = 1) were entered as continuous variables into multivariable linear regression models to evaluate linear trends per 1-SD increase; this served as the primary analytical strategy. The standardized dietary pattern factor scores were also categorized into quartiles (Q1, Q2, Q3, Q4) using the percentile method, with Q1 representing the lowest adherence group and Q4 the highest adherence group, to perform supplementary between-group comparisons and visually illustrate effect differences across varying levels of adherence as well as potential non-linear patterns. Quartile-based comparisons were used solely as a descriptive supplementary analysis and did not constitute the primary inferential framework; the primary basis for inference was the trend test using standardized continuous scores.

Multiple linear regression was used to examine the associations between pre-pregnancy dietary patterns and children’s body composition. In the multivariable linear regression models, each outcome variable (PBF, FMI, FFMI, SMI) was included as a continuous variable, and the regression coefficient (β) represented the absolute unit change in the respective body composition indicator (PBF: %; FMI: kg/m^2^; FFMI: kg/m^2^; SMI: kg/m^2^). Multicollinearity was assessed in all models; the variance inflation factor (VIF) for all variables was <2, indicating no substantial multicollinearity. Potential confounders were identified through univariate screening (*p* < 0.20) from a pool of covariates including sociodemographic characteristics, birth information, feeding practices, dietary intake, physical activity, and illness status at ages 3–6 years. Two models were constructed: a crude model (unadjusted) and a multivariable model adjusted for maternal age, pre-pregnancy BMI, education level, household income, parity, and child sex, age, birth weight, birth length, feeding mode, secondhand smoke exposure during pregnancy, gestational weight gain, paternal BMI, nutritional supplement use during pregnancy, gestational diabetes mellitus, and pregnancy-induced hypertension.

Standard regression diagnostics were performed for all multivariable linear models across the four body composition outcomes. Multicollinearity was assessed by variance inflation factor (VIF); all values were below 2.0 (range 1.03–1.81), indicating no serious multicollinearity. Normality of residuals was examined using histograms and normal P–P plots, which showed no substantial departure from normality. Homoscedasticity and linearity were evaluated by plotting standardized residuals against standardized predicted values; the scatterplots displayed a random, horizontal band around zero with no discernible funnel shape or systematic curvature. Influential observations were identified using Cook’s distance; no observations exceeded the conventional threshold of 1.0 (maximum Cook’s distance < 0.22 across all models), indicating that the regression estimates were not unduly driven by single data points. For the FMI and SMI models, a small number of observations had extreme standardized residuals (>3); however, their Cook’s distances were well below 1.0, and sensitivity analyses excluding these extreme residuals yielded materially unchanged effect estimates, confirming the robustness of the main findings. Representative diagnostic plots are provided in Supplementary Figure S2

Results are presented as unstandardized regression coefficients (β) with 95% confidence intervals (CI). Effect modification was assessed by introducing interaction terms between each dietary pattern (as a standardized continuous variable, i.e., factor score) and stratification variables into the multivariable model. Stratified analyses were conducted by offspring sex (male/female) and maternal pre-pregnancy BMI (<24 kg/m^2^ and ≥24 kg/m^2^), with crude and multivariable models fitted separately in each subgroup. Dose–response relationships were evaluated by entering standardized dietary pattern factor scores as continuous variables into the regression models; *p* values for trend are reported per 1-standard deviation (SD) increase. Quartile ranks, or median values, were not used as the basis for trend tests. All tests were two-sided, with a significance level of α = 0.05.

## 3. Results

### 3.1. Basic Information and Follow-Up Status of Mothers and Preschool Children, and Paternal Information

This study included 582 mother–child pairs; the baseline characteristics of mothers and preschool children are presented in Table 1. Nearly half of the mothers were aged 25–29 years (49.8%), with a mean height of 159.68 ± 5.10 cm. Pre-pregnancy BMI was predominantly within the normal range (58.1%), whereas overweight, obesity, and underweight accounted for 22.9%, 13.1%, and 6.0%, respectively. Household monthly income was primarily between ¥2000 and ¥3000 (41.9%), and educational attainment was mostly at junior high school level or below (64.3%). The majority engaged in light physical labor (57.9%), and primiparas constituted 72.2%. The male-to-female ratio among infants was balanced. Most were full-term infants (gestational age ≥ 39 weeks, 77.1%), with a mean birth length of 50.06 ± 0.99 cm and a mean birth weight of 3396.54 ± 449.28 g. Exclusive breastfeeding accounted for 74.20%, while formula feeding accounted for 25.80%. Secondhand smoke exposure during pregnancy was reported by 29.7% of the participants. The reported prevalence of gestational diabetes mellitus was low at 1.9%, with an additional 15.1% reporting uncertainty; the reported prevalence of pregnancy-induced hypertension was 14.8%, with 24.7% reporting uncertainty. Mean gestational weight gain was 15.67 ± 5.43 kg. Regarding lifestyle and nutritional supplementation, 63.1% of the participants reported using nutritional supplements during pregnancy. The reported rates of smoking and alcohol consumption within the six months prior to pregnancy were extremely low at 0.7% and 0.9%, respectively. Paternal BMI was predominantly in the overweight category (43.6%), followed by normal weight (34.5%) and obesity (19.1%), with underweight accounting for only 2.7%. Paternal education was predominantly junior high school or below (66.5%), with senior high school/vocational high school/technical secondary school accounting for 14.1% and college or above accounting for 19.4%.

The mean age of the preschool children was 4.43 ± 1.11 years, with a mean height of 105.95 ± 6.92 cm and mean weight of 17.48 ± 3.03 kg; the mean BMI was 15.49 ± 1.53 kg/m^2^. Regarding body composition, the mean percentage of body fat (PBF) was 16.70 ± 5.64%, fat mass index (FMI) was 2.66 ± 1.18 kg/m^2^, fat-free mass index (FFMI) was 12.86 ± 0.74 kg/m^2^, and skeletal muscle mass index (SMI) was 4.11 ± 0.70 kg/m^2^. During follow-up (ages 3–6 years), children’s daily diet primarily consisted of grains (73.32 ± 30.87 g/day), light-colored vegetables (66.24 ± 37.23 g/day), fruits (86.10 ± 43.06 g/day), and dairy products (71.15 ± 53.79 g/day); meat and egg intake was 43.68 ± 22.51 g/day and 25.14 ± 12.94 g/day, respectively. Intakes of legumes, fungi/algae, nuts, and beverages were all low (<5 g/day). The median daily water intake was 1115 mL (IQR: 882–1290). Nearly half of the children (46.9%) took nutritional supplements. Children engaged in an average of 2.91 ± 1.20 h of indoor activities and 2.65 ± 0.79 h of outdoor activities daily, with prolonged sitting time of 2.27 ± 0.85 h and screen time of 1.41 ± 0.74 h, while average sleep duration was 11.63 ± 1.07 h. Within the past month, 26.5% reported experiencing cold or fever, whereas the incidence rates of diarrhea and pneumonia were relatively low (0.9% and 3.1%, respectively).

### 3.2. Dietary Pattern Extraction

The Kaiser-Meyer-Olkin (KMO) test and Bartlett’s test of sphericity (KMO = 0.777; Bartlett’s *p* < 0.001) indicated that the data were suitable for principal component analysis (PCA). Based on the criteria of eigenvalues > 1.0, the inflection point of the scree plot, and the rotated factor loading matrix, five principal components were extracted, accounting for a cumulative variance contribution rate of 52.79%. The scree plot is presented in Figure S1 of the Supplementary Materials. Food groups with factor loadings > 0.30 were retained in each pattern. These components were: Animal-based food pattern (22.81%, dominated by fish, poultry, and eggs); grain-vegetable-fruit pattern (11.06%); processed meat-dairy pattern (7.53%); vegetable-legume pattern (6.61%); and Cured vegetable pattern (6.44%). The complete rotated factor loading matrix, including the loadings of all 16 food groups on the five components (including those with absolute values < 0.30), is presented in Table 2 in the main text and in Table S1 in the Supplementary Materials. The communalities of the food groups ranged from 0.249 to 0.850, indicating that the extracted components explained moderate to good proportions of the variance in each food group.

### 3.3. Analysis of the Correlation Between Maternal Dietary Patterns Before Pregnancy and Body Composition in Preschool-Aged Children

Table 3 presents the primary trend analyses based on standardized continuous dietary pattern scores (per 1-SD increase), alongside supplementary between-group comparisons across quartiles (Q1–Q4) for descriptive purposes. Multivariable linear regression analyses revealed that the associations between the five maternal pre-pregnancy dietary patterns and offspring body composition differed in a pattern-specific manner. After multivariable adjustment, the processed meat-dairy pattern was positively associated with higher FMI; the vegetable-legume pattern was associated with both higher PBF and lower SMI; the grain-vegetable-fruit pattern was inversely associated with FFMI and SMI; the animal-based food pattern showed positive trends with PBF and FMI in the crude model but lost significance after adjustment for confounders; and the cured vegetable pattern showed only a limited association with FFMI.

Specifically, for the animal-based food pattern (Pattern 1), in the crude model, positive linear trends were observed for PBF (*p* for trend = 0.01) and FMI (*p* for trend = 0.013). However, after multivariable adjustment, neither the β coefficients for Q2–Q4 nor the trend tests reached statistical significance (PBF: *p* for trend = 0.177; FMI: *p* for trend = 0.199), suggesting that the associations of this pattern with offspring adiposity indicators may be explained by confounding factors. For the grain-vegetable-fruit pattern (Pattern 2), in the multivariable-adjusted model, compared with Q1, participants in Q2 to Q4 had lower FFMI (Q4:β = −0.265,95% CI: −0.510 to −0.020) and SMI (Q4: β = −0.256, 95% CI: −0.510 to −0.002). A significant linear trend was observed for SMI (*p* for trend = 0.012) based on the standardized continuous score, while the trend for FFMI was marginally significant (*p* for trend = 0.059). No significant associations were observed for PBF or FMI. For the processed meat-dairy pattern (Pattern 3), in the multivariable-adjusted model, FMI in Q4 was significantly higher than that in Q1 (β = 0.262, 95% CI: 0.036–0.488), and PBF also showed an increasing trend (β = 0.215, *p* for trend < 0.01). No significant associations were observed for FFMI or SMI. For the vegetable-legume pattern (Pattern 4), in the multivariable-adjusted model, PBF in Q3 was significantly higher than that in Q1 (β = 0.234, 95% CI: 0.007–0.461); SMI in Q4 was significantly lower than that in Q1 (β = −0.338, 95% CI: −0.564 to −0.113), with a significant linear trend based on the standardized continuous score (*p* for trend = 0.001). FFMI showed inverse trends across Q2–Q4 (Q4: β = −0.206, 95% CI: −0.423 to 0.011), although the difference did not reach statistical significance. This pattern was associated with both adiposity and muscle indices. For the cured vegetable pattern (Pattern 5), in the multivariable-adjusted model, only FFMI in Q3 was significantly higher than that in Q1 (β = 0.257, 95% CI: 0.033–0.481). No significant associations were observed for PBF, FMI, or SMI, nor were linear trends detected.

### 3.4. Analysis of Gender-Stratified Results

**Figure 2:**
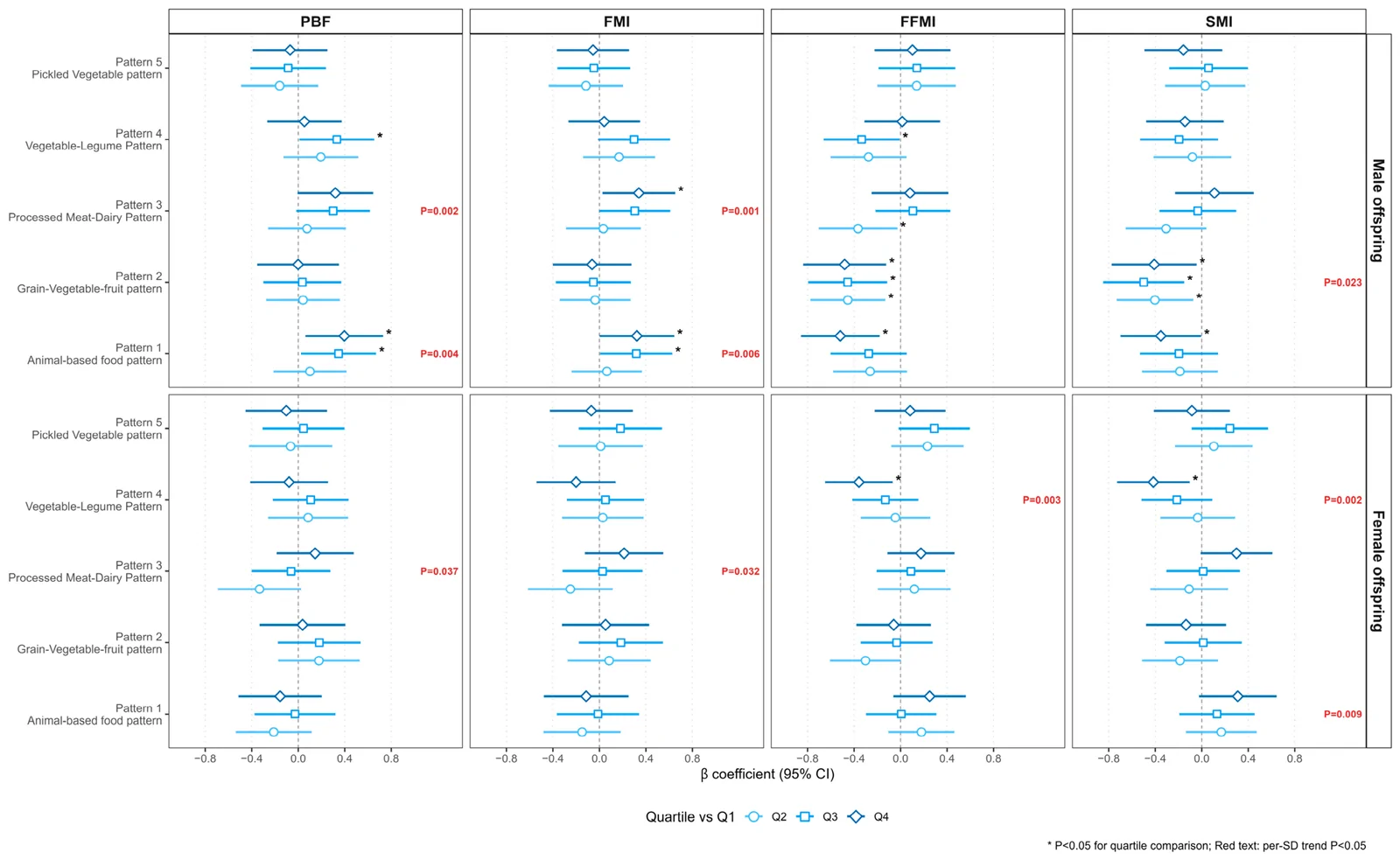
Associations between maternal pre-pregnancy dietary patterns and offspring body composition by offspring sex (Multivariable−adjusted model).

The results of sex-stratified analyses are presented in Figure 2 and Table S2. To account for multiple comparisons, Benjamini–Hochberg false discovery rate (FDR) correction was applied within the family of 20 sex-interaction tests (Table S3). These subgroup analyses are exploratory in nature; claims of effect modification are supported only where interaction tests reach statistical significance after FDR correction. Interaction tests revealed nominally significant modification by offspring sex on the associations of the animal-based food pattern with FFMI and SMI (nominal *p* for interaction = 0.048 and 0.013, respectively); however, neither survived FDR correction (FDR-adjusted *p* = 0.480 and 0.260, respectively). Therefore, these apparent sex differences should be interpreted cautiously as exploratory findings rather than evidence of true effect modification. No significant effect modification by sex was observed for the associations of the animal-based food pattern with PBF or FMI (nominal *p* for interaction = 0.173 and 0.317, respectively), nor for any other dietary pattern with any body composition outcome (all FDR-adjusted *p* > 0.05).

Among males, the animal-based food pattern was positively associated with PBF and FMI: compared with Q1, the β coefficients for Q4 were 0.396 (95% CI: 0.062, 0.730) and 0.322 (95% CI: 0.001, 0.644), with *p* for trend = 0.004 and 0.006, respectively; meanwhile, FFMI in Q4 was significantly lower than that in Q1 (β = −0.519, 95% CI: −0.857, −0.180). The grain-vegetable-fruit pattern was associated with lower FFMI and SMI: compared with Q1, participants in Q2 to Q4 had lower FFMI (Q4: β = −0.481, 95% CI: −0.838, −0.124) and SMI (Q4: β = −0.409, 95% CI: −0.775, −0.044), with a significant trend for SMI (*p* for trend = 0.023) and a marginally significant trend for FFMI (*p* for trend = 0.203). No significant associations were observed for PBF or FMI. The processed meat–dairy pattern was positively associated with higher PBF and FMI (*p* for trend = 0.002 and 0.001, respectively). For the vegetable-legume pattern, PBF (β = 0.332, 95% CI: 0.010, 0.653) and FMI (β = 0.298, 95% CI: −0.012, 0.608) in Q3 were positively associated, whereas FFMI in Q3 was inversely associated (β = −0.335, 95% CI: −0.662, −0.008); SMI showed inverse trends across Q2–Q4, although none reached statistical significance. The cured vegetable pattern showed no significant associations in the multivariable-adjusted model.

Among females, the animal-based food pattern showed a positive trend with SMI (*p* for trend = 0.009), although the differences between quartile groups were not statistically significant (Q4: β = 0.310, 95% CI: −0.024, 0.644); the association with FFMI was marginally significant (*p* for trend = 0.068). The processed meat-dairy pattern was positively associated with both PBF and FMI (*p* for trend = 0.037 and 0.032, respectively). For the vegetable-legume pattern, FFMI (β = −0.358, 95% CI: −0.649, −0.068) and SMI (β = −0.416, 95% CI: −0.728, −0.104) in Q4 were significantly lower than those in Q1, with significant linear trends for both (*p* for trend = 0.003 and 0.002, respectively). The grain–vegetable–fruit pattern and the cured vegetable pattern showed no significant associations in the multivariable-adjusted model.

For all sex-stratified findings, including those for the animal-based pattern, results are presented for exploratory purposes only, as none of the interaction tests survived FDR correction. In particular, the associations of plant-based dietary patterns (the grain-vegetable-fruit pattern and the vegetable-legume pattern) with lower muscle indices may reflect overall dietary characteristics—such as differences in total protein or energy intake—and should not be overinterpreted as negative effects of plant-based diets on childhood body composition. Furthermore, the observed sex differences may be influenced by prepubertal sex-specific growth patterns, physical activity levels, and metabolic differences, and the underlying mechanisms warrant further investigation.

### 3.5. Analysis of Pre-Pregnancy Body Mass Index-Stratified Results

**Figure 3:**
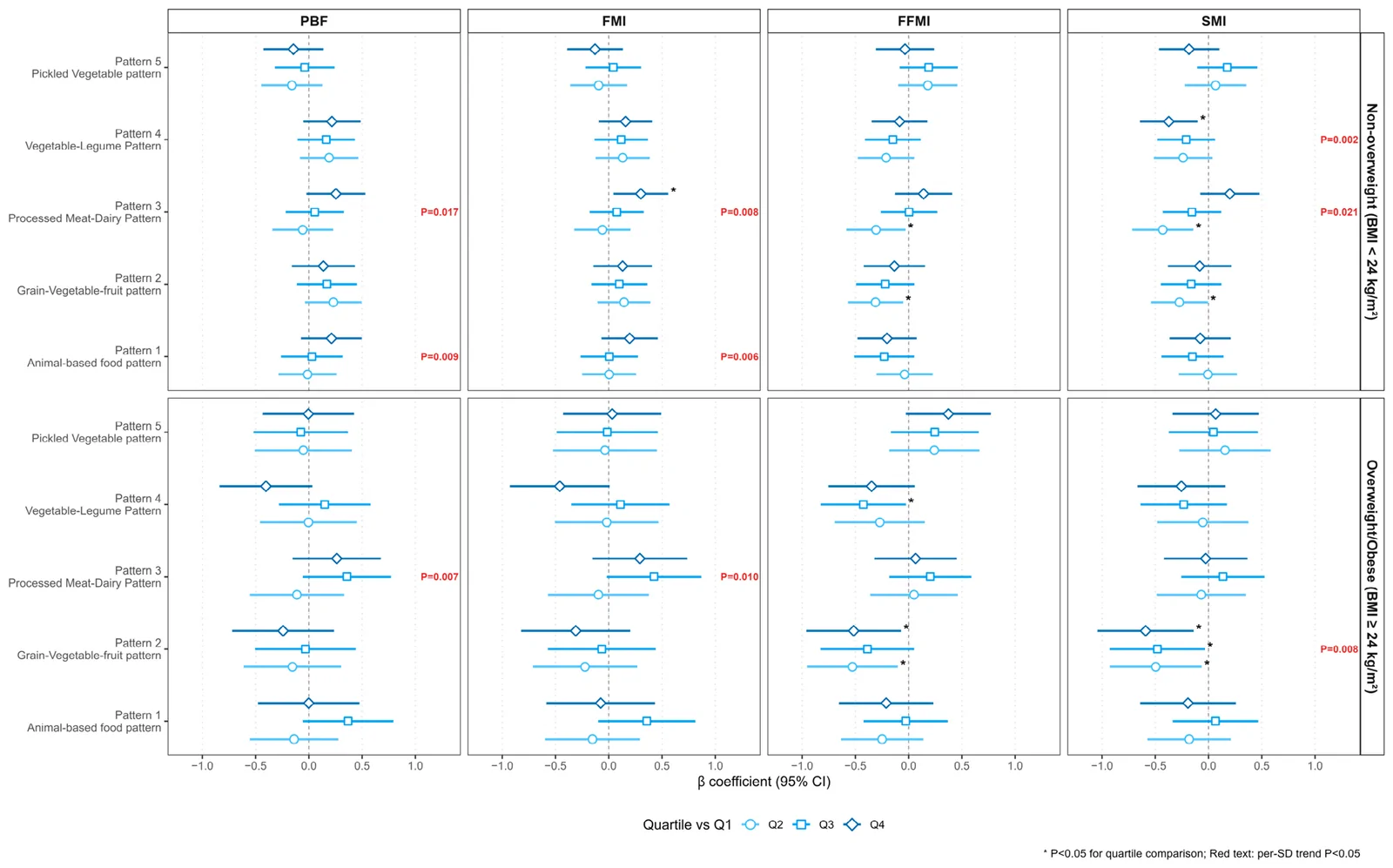
Associations between maternal pre−pregnancy dietary patterns and offspring body composition by maternal pre−pregnancy BMI (Multivariable−adjusted model).

The results of analyses stratified by maternal pre-pregnancy BMI are presented in Figure 3 and Table S2. To account for multiple comparisons, Benjamini–Hochberg FDR correction was applied within the family of 20 BMI-interaction tests (Table S3). These subgroup analyses are exploratory in nature; claims of effect modification are supported only where interaction tests reach statistical significance after FDR correction. Interaction tests revealed nominally significant modification by maternal pre-pregnancy BMI on the association between the processed meat-dairy pattern and SMI (nominal *p* for interaction = 0.033); however, this did not survive FDR correction (FDR-adjusted *p* = 0.502). Therefore, this apparent BMI modification should be interpreted cautiously as an exploratory finding rather than evidence of true differential effects. No significant effect modification was observed for other dietary patterns with any body composition outcome (all FDR-adjusted *p* > 0.05).

In the non-overweight/non-obese group (BMI < 24 kg/m^2^), the animal-based food pattern was positively associated with higher PBF and FMI (*p* for trend = 0.009 and 0.006, respectively). The grain-vegetable-fruit pattern was associated with lower FFMI and SMI: FFMI (β = −0.311, 95% CI: −0.570, −0.052) and SMI (β = −0.273, 95% CI: −0.541, −0.005) in Q2 were significantly lower than those in Q1, although no significant linear trend was observed across quartile groups. The processed meat-dairy pattern was positively associated with higher PBF (*p* for trend = 0.017) and FMI (*p* for trend = 0.008), and inversely associated with lower SMI (*p* for trend = 0.021). For the vegetable-legume pattern, SMI in Q4 was significantly lower than that in Q1 (β = −0.373, 95% CI: −0.644, −0.102), with a significant linear trend (*p* for trend = 0.002). The cured vegetable pattern showed no significant associations in the multivariable-adjusted model.

In the overweight/obese group (BMI ≥ 24 kg/m^2^), no significant differences were observed across quartile groups for the animal-based food pattern. The grain-vegetable-fruit pattern was associated with lower FFMI and SMI: FFMI (β = −0.528, 95% CI: −0.953, −0.102) and SMI (β = −0.496, 95% CI: −0.927, −0.064) in Q2 were significantly lower than those in Q1, and SMI in Q4 was also significantly reduced (β = −0.591, 95% CI: −1.043, −0.140), with a significant linear trend for SMI (*p* for trend = 0.008). The processed meat–dairy pattern was positively associated with higher PBF (*p* for trend = 0.007) and FMI (*p* for trend = 0.010). For the vegetable-legume pattern, PBF (β = −0.403, 95% CI: −0.839, 0.033) and FMI (β = −0.460, 95% CI: −0.928, 0.008) in Q4 showed decreasing trends, although neither reached statistical significance. For the cured vegetable pattern, FFMI in Q4 showed an increasing trend (β = 0.373, 95% CI: −0.026, 0.772), but it did not reach statistical significance.

For all BMI-stratified findings, including those for the processed meat–dairy pattern with SMI, results are presented for exploratory purposes only, as none of the interaction tests survived FDR correction. The more pronounced inverse association between the processed meat-dairy pattern and SMI among children of overweight/obese mothers warrants further validation regarding its clinical significance. Moreover, the associations of plant-based dietary patterns (the grain-vegetable-fruit pattern and the vegetable-legume pattern) with lower muscle indices may reflect overall dietary characteristics—such as differences in total protein or energy intake—and should not be overinterpreted as negative effects of plant-based diets on childhood body composition.

## 4. Discussion

This study identified five distinct preconception dietary patterns and examined their associations with body composition indicators in preschool-aged children. To our knowledge, this is among the few studies in China to specifically investigate the relationship between maternal pre-pregnancy dietary patterns and body composition in preschool children, as well as the potential modifying effects of offspring sex and maternal pre-pregnancy body mass index (BMI). These findings are situated within the broader literature on the Developmental Origins of Health and Disease (DOHaD), which has increasingly recognized the preconception period as a critical stage for understanding early nutritional influences on offspring development. Research by Huang et al. has shown that adult pathogenesis may be associated with epigenetic changes during gametogenesis and early embryonic development, establishing links among preconception, embryonic, fetal, and early infant stages and the occurrence of disease in adulthood [32]. Recent animal studies further reveal that a maternal high-fat diet before embryo implantation can alter uterine microRNA expression, thereby influencing the metabolic health of offspring in adulthood [33]. These findings suggest that the preconception period may be an important stage for understanding early nutritional influences on offspring development; however, the present study is observational and unable to directly verify underlying biological mechanisms, and confirmation through biomarker integration in future research is warranted.

Currently, most studies on maternal diet and offspring body composition focus on the prenatal period, whereas specialized epidemiological studies targeting the preconception period remain relatively scarce. In fact, the preconception and periconceptional stages exhibit significant overlap in developmental sensitivity, with both phases being highly responsive to maternal nutritional status [28]. The potential influence of parental pre-pregnancy nutrition on offspring phenotype and metabolic syndrome risk has also been systematically reviewed [34]. Maternal pre-pregnancy nutrition may be associated with the metabolic characteristics of offspring through epigenetic mechanisms (including DNA methylation, histone modifications, and non-coding RNAs) [34]. By examining maternal pre-pregnancy diet, this study extends existing evidence from the prenatal to the pre-pregnancy stage, though the observed associations do not imply causal programming effects.

We found that the animal-based food pattern showed positive trends with percentage of body fat (PBF) and fat mass index (FMI) in preschool children in the crude model; however, these associations did not reach statistical significance after multivariable adjustment, suggesting that the observed effects were largely explained by confounding factors. In sex-stratified analyses, this pattern was associated with higher PBF and FMI and lower FFMI in boys, whereas it was associated with higher FFMI and SMI in girls, with interaction tests revealing nominally significant modification by offspring sex (nominal *p* for interaction = 0.048 and 0.013 for FFMI and SMI, respectively); however, neither survived Benjamini–Hochberg false discovery rate (FDR) correction within the family of 20 sex-interaction tests (FDR-adjusted *p* = 0.480 and 0.260, respectively). Therefore, these apparent sex differences should be interpreted cautiously as exploratory findings rather than evidence of true effect modification. Nevertheless, maternal pre-pregnancy BMI did not significantly affect these associations.

It should be noted that human studies directly evaluating the impact of pre-pregnancy animal-based food intake on offspring body composition are extremely scarce in the current literature. Given the high continuity between pre-pregnancy and gestational diets, and the fact that the periconceptional period constitutes a shared window for fetal developmental programming, the following prenatal studies may serve as indirect references. For instance, Madsen et al. reported that each 5% increase in maternal protein intake during pregnancy was linearly associated with increased risks of overweight and obesity in children at 7 and 11 years of age [17]. Similarly, a Danish birth cohort study demonstrated that adherence to an animal-based, low-glycemic-index diet during pregnancy was significantly associated with higher BMI in offspring at 18 years of age [35].

In contrast to the aforementioned Western cohorts, the animal-based pattern in our study did not reach statistical significance after full adjustment. This discrepancy may be attributable to differences in protein sources: Western cohorts predominantly derive animal protein from red meat and dairy products, whereas in our cohort, fish, shrimp, and poultry showed higher factor loadings (>0.68), with a relatively lower contribution of red meat. Moreover, the absolute intake of animal-based foods among rural Chinese residents is generally lower than that in Western populations, which may result in smaller effect sizes. Differences in overall energy intake and dietary structure between populations may also contribute to this divergence.

Of particular note is the statistical association between plant-based dietary patterns—especially the grain-vegetable-fruit pattern and the vegetable-legume pattern—with lower FFMI and SMI in offspring. This finding warrants cautious interpretation for several reasons. First, given the observational nature of this study, causality cannot be inferred, and residual confounding cannot be ruled out. Second, as the present analysis was based on food-group-derived dietary patterns without direct adjustment for total energy intake or assessment of specific nutrient intakes (e.g., protein, amino acids), we are unable to determine whether this association is attributable to the high proportion of plant-based foods itself or to concomitant insufficient total protein or energy intake. Third, plant-based dietary patterns in certain populations may be associated with lower socioeconomic status, poorer dietary diversity, or lower overall diet quality, all of which may influence childhood body composition through various pathways. We were unable to fully capture these socioeconomic gradients; therefore, residual confounding cannot be excluded.

Chen et al. reported that higher dietary quality during pregnancy was associated with lower fat mass index in offspring, although the impact of plant-based diets on muscle development remains unclear [14]. The association between plant-based patterns and lower muscle indices observed in our study is not inconsistent with Chen’s findings, and may instead reflect the specific composition of plant-based diets in rural Chinese settings: the “grain-vegetable-fruit” pattern in this cohort is dominated by refined grains and commonly consumed vegetables, with low intake of nuts and whole grains. The “vegetable-legume” pattern, although rich in dark vegetables and soy products, is characterized by legume intake predominantly in processed forms such as tofu and soy milk, and often coexists with pickled vegetables and high-salt cooking practices. This composition is fundamentally different from the “Mediterranean-style” plant-based diet in Western contexts, which is abundant in legumes, nuts, and whole grains. In studies conducted in rural Chinese populations, plant-based diets are rich in fiber and micronutrients, but may be relatively deficient in high-quality protein or specific branched-chain amino acids required for fetal muscle development [36]. The observed association between the grain-vegetable-fruit combination pattern and lower lean mass indices in boys and children born to overweight/obese mothers further suggests that the metabolic environment resulting from maternal obesity may interact with plant-based diets, thereby influencing the body composition of offspring [29]. In stratified analyses, the grain-vegetable-fruit pattern appeared to be associated with lower lean mass indices in boys and in children of overweight/obese mothers; however, these subgroup findings were not supported by significant interaction tests and should be considered exploratory. Whether maternal obesity status interacts with dietary patterns to influence offspring body composition cannot be determined from this observational study and requires further investigation.

It should be emphasized that the present findings should not be interpreted as evidence of adverse effects of plant-based diets on childhood muscle development. Vegetables, fruits, and legumes are rich in dietary fiber, vitamins, minerals, and phytochemicals, and confer well-established benefits for both maternal and offspring health. Our findings merely suggest that, at the level of overall dietary patterns, a pre-pregnancy diet dominated by plant-based foods with potentially inadequate animal-source food intake is statistically associated with childhood muscle indices. The biological mechanisms and clinical significance of this association require further validation in studies that adequately control for nutrient intakes, including total energy and protein.

The consumption pattern characterized by high intake of processed foods and animal-derived products, particularly processed meats and dairy products, exhibited a positive correlation with the offspring’s fat mass index (FMI) and body fat percentage (PBF). This finding aligns with the study by Wang et al., which demonstrated in a cohort of nearly 20,000 mother–child pairs that maternal consumption of highly processed foods increased the risk of overweight or obesity in offspring by 26% [13]. Although Wang et al. evaluated dietary patterns during pregnancy, there is continuity in exposure characteristics between pre-pregnancy and pregnancy: such dietary patterns typically feature high energy density, abundant saturated fats and refined carbohydrates, and insufficient fiber.

Of note, the term “processed meat” in the Chinese context encompasses preserved or smoked products such as sausages, cured meat, and ham, which are high in sodium and nitrite content, and thus differ nutritionally from processed meats in Western settings—which are predominantly cold cuts and hamburger patties. Furthermore, the “cured vegetable” pattern in this study independently explained 6.44% of the variance, reflecting the widespread consumption of traditional high-salt preserved vegetables in rural Chinese populations—a dietary component that typically does not emerge as an independent pattern in Western cohorts. These cultural specificities may influence the direction and magnitude of the observed associations. From a biological perspective, these dietary patterns—characterized by high energy density, rich saturated fats and refined carbohydrates, and low fiber content—have been hypothesized in experimental studies to influence maternal metabolic status; however, our observational data do not permit inference regarding specific biological pathways [37].

Our results were consistent across sexes, and interaction tests indicated no significant modifying effect of offspring sex. Although the point estimates were larger in non-overweight mothers, no BMI interaction tests survived FDR correction for multiple comparisons. Although the processed meat-dairy pattern with SMI showed nominal significance (nominal *p* for interaction = 0.033), this did not survive Benjamini–Hochberg FDR correction within the family of 20 BMI-interaction tests (FDR-adjusted *p* = 0.502).; therefore, any subgroup differences are likely attributable to variations in sample size or random fluctuation.

Although the observed associations between pre-pregnancy dietary patterns and offspring body composition persisted after adjustment for measured confounders, this should not be interpreted as evidence that they are independent of broader lifestyle factors. Multivariable adjustment reduces confounding by measured variables, but it does not demonstrate that these associations are independent of unmeasured factors such as maternal energy intake, stress, paternal diet, gestational diet, and other familial characteristics. A more appropriate interpretation is that these associations remain after controlling for the measured variables included in our models.

A notable observation in this study was the presence of sex differences in the association between the animal-based dietary pattern and offspring body composition. Nominally significant modification by offspring sex was observed for the associations of this pattern with FFMI and SMI (nominal *p* for interaction = 0.048 and 0.013, respectively); however, neither survived Benjamini–Hochberg FDR correction within the family of 20 sex-interaction tests (FDR-adjusted *p* = 0.480 and 0.260, respectively). Therefore, these apparent sex differences should be interpreted cautiously as exploratory findings rather than evidence of true effect modification by sex. For other dietary patterns, although point estimates varied between sexes in stratified analyses, interaction tests did not reach statistical significance; therefore, these apparent differences should be interpreted cautiously as exploratory findings rather than evidence of true effect modification by sex. These findings are consistent with accumulating evidence from animal models [21] and human cohorts [22] suggesting that maternal nutritional exposures may be differentially associated with offspring body composition by sex. For example, Campbell et al. observed that maternal high-fat, high-sugar diet exposure was associated with greater post-weaning fat mass in male offspring, whereas female offspring showed distinct patterns of lean mass accumulation [21]. Similarly, periconceptional high-fat diet exposure has been associated with sex-specific cardiovascular and immune adaptations in animal models [38]. The mechanisms underlying such sex differences remain unclear and may involve differences in placental adaptation and sex hormone-mediated biological pathways [32,33], though our study did not directly assess these pathways.

It is important to acknowledge the issue of multiple comparisons in this study. We examined associations between five dietary patterns and four body composition outcomes (20 tests in total), in addition to subgroup and interaction analyses. Using the Benjamini–Hochberg FDR correction for three predefined hypothesis families—20 main-effect tests, 20 sex-interaction tests, and 20 BMI-interaction tests—only five main-effect associations remained statistically significant (processed meat-dairy pattern with PBF and FMI, and vegetable-legume pattern with SMI). None of the interaction tests survived FDR correction. Therefore, claims of effect modification in this study are not supported after correction for multiple testing. For all dietary patterns, although point estimates varied between sexes or BMI subgroups in stratified analyses, the corresponding interaction tests were not statistically significant after FDR correction. These subgroup findings are presented for exploratory and descriptive purposes only and should not be interpreted as evidence of differential effects between subgroups.

Regarding the potential modifying effect of maternal pre-pregnancy BMI, a nominally significant interaction was observed for the association between the processed meat-dairy pattern and SMI (nominal *p* for interaction = 0.033); however, this did not survive FDR correction (FDR-adjusted *p* = 0.502). Therefore, this apparent BMI modification should be interpreted cautiously as an exploratory finding. For all other dietary patterns and body composition outcomes, the FDR-adjusted *p* values for interaction were > 0.05. Nevertheless, pre-pregnancy BMI itself remained a significant independent predictor of these outcomes, a finding consistent with previous studies that have consistently identified maternal pre-pregnancy BMI as a strong independent predictor of offspring body composition and adiposity [39,40,41].

Regarding the potential biological mechanisms, it should be clearly noted that we did not measure any inflammatory markers, insulin sensitivity indices, epigenetic modifications, or gut microbiota indicators in this study; therefore, we are unable to verify specific molecular pathways. The mechanisms previously discussed in the literature—including chronic low-grade inflammation, insulin and glucose homeostasis dysregulation, and epigenetic modifications such as DNA methylation—are reasonable scientific hypotheses derived from animal experiments or adult studies, rather than conclusions directly supported by our data. We suggest that these mechanisms be considered solely as testable directions for future research, rather than as definitive explanations for the findings of the present study.

The strengths of this study include its prospective design, the specific evaluation of maternal diet during the pre-pregnancy (rather than pregnancy) period, the use of principal component analysis to identify data-driven dietary patterns, and the comprehensive assessment of offspring body composition—including both adiposity and muscle indices.

This study has several limitations. In early pregnancy, women were asked to recall their habitual diet during the month before pregnancy, which may have introduced recall bias and measurement error. Although we adjusted for multiple potential confounders—including maternal age, pre-pregnancy BMI, parity, family income, parental education, paternal BMI, gestational weight gain, use of dietary supplements, gestational diabetes mellitus, and gestational hypertensive disorders—we did not assess total energy intake, nor did we measure other potential confounders such as maternal psychological stress or paternal diet, leaving the possibility of residual confounding. The observed associations between pre-conception dietary patterns and offspring body composition may also partially reflect lifestyle or socioeconomic factors already present before conception that have continued to influence offspring. Therefore, we cannot exclude the possibility of residual confounding from unmeasured or poorly measured variables, and we do not claim that these associations are independent of broader lifestyle factors. Pre-pregnancy BMI was calculated based on self-reported pre-pregnancy weight, which may be subject to recall bias. However, previous studies conducted in impoverished rural areas of China have shown that the mean error of self-reported pre-pregnancy weight is generally within ±1–2 kg, suggesting that the impact on BMI classification is likely limited [42].

The follow-up completion rate was 77.4% (605/782), calculated based on singleton live births eligible for preschool follow-up. Among the 177 children not included in the preschool follow-up, the majority were lost due to external factors such as family relocation or not having reached the minimum kindergarten enrollment age (3 years) at the time of follow-up; these reasons are unlikely to be directly associated with maternal pre-pregnancy dietary exposures or baseline health indicators. However, because outcome data are completely missing for these participants, we were unable to statistically test whether the missing mechanism was non-random (MNAR). We therefore deliberately did not present formal baseline comparisons between included and excluded groups in the results, as such comparisons could misleadingly imply absence of bias even when MNAR cannot be ruled out. Nevertheless, selective attrition cannot be fully excluded as a source of bias potentially affecting the generalizability of our findings.

For the final analytic sample (*n* = 582), item-level missing data were minimal (body composition: *n* = 6; questionnaire items: *n* = 17; combined <4%). We used complete-case analysis, as the low proportion of missingness minimized the risk of bias, and multiple imputation was not performed because the missing-at-random assumption could not be adequately verified and imputation models lacked sufficient outcome-related predictors.

The sample was drawn from a single rural region, which may limit generalizability to urban or other ethnic populations. In addition, dietary intake during pregnancy and the early postpartum period was not systematically tracked, precluding the ability to rule out confounding or effect modification by changes in maternal diet during these periods. Although preschool-aged children’s dietary data were collected using an FFQ and weighted to combine information from inside and outside kindergarten, intakes of individual food groups did not meet the inclusion threshold (*p* < 0.20) in univariate screening for association with body composition outcomes. Moreover, because condiments such as oil, salt, and sugar were not surveyed, total energy intake could not be estimated; consequently, children’s dietary data were not included in the multivariable models, and formal mediation analyses were not performed. Additionally, the associations between plant-based dietary patterns and muscle indices are examined without a strong a priori directional hypothesis and should be considered exploratory; these findings are hypothesis-generating and should not be interpreted as evidence of adverse effects of plant-based diets per se. Nominally significant effect modification was observed for offspring sex (animal-based pattern with FFMI and SMI) and maternal pre-pregnancy BMI (processed meat–dairy pattern with SMI). However, none of these interactions survived Benjamini–Hochberg FDR correction (FDR-adjusted *p* = 0.260–0.502). We therefore emphasize effect sizes and confidence intervals over statistical significance, and recommend that these findings be validated in independent samples. Future studies should employ prospective cohort designs, integrate biomarker validation, and incorporate family-level dietary assessments to further elucidate the mechanisms linking pre-pregnancy diet to offspring body composition.

## 5. Conclusions

In this cohort of Chinese rural women of childbearing age, we observed selective associations between maternal pre-pregnancy dietary patterns and body composition indicators in preschool children. Specifically, after multivariable adjustment, the processed meat-dairy pattern was associated with higher fat mass index (FMI); the vegetable-legume pattern was associated with lower skeletal muscle index (SMI); and the grain-vegetable-fruit pattern was inversely associated with both fat-free mass index (FFMI) and SMI. These associations persisted after adjustment for known confounders, but given the observational nature of this study, they should not be interpreted as evidence that the pre-pregnancy period represents a modifiable window for influencing offspring body composition.

Furthermore, although the animal-based food pattern showed opposite directions of association with FFMI and SMI across sexes in stratified analyses, and interaction tests revealed nominal significance (nominal *p* for interaction = 0.048 and 0.013, respectively), neither survived FDR correction (FDR-adjusted *p* = 0.480 and 0.260, respectively). Therefore, these findings should be interpreted as exploratory and do not support differential dietary recommendations stratified by sex.

Although a nominally significant BMI modification was observed for the association between the processed meat-dairy pattern and SMI (nominal *p* for interaction = 0.033), this did not survive FDR correction (FDR-adjusted *p* = 0.502). Therefore, these results should not be taken as evidence that dietary pattern associations are modified by maternal obesity-related metabolic factors.

Finally, the associations of plant-based dietary patterns (the grain-vegetable-fruit and vegetable-legume patterns) with lower muscle indices likely reflect characteristics of the overall dietary structure, such as protein quantity or quality, rather than adverse effects of plant-based foods per se. All findings are observational and cannot establish causality, nor can the influence of unmeasured confounders—including total energy intake, physical activity, and socioeconomic status—be excluded. Further validation in studies with more precise nutritional assessment and in interventional designs is warranted.

## Figures and Tables

**Table 1:** Basic Information and Follow-up Status of Mothers and Preschool Children [*n* (%) = 582].

Characteristics	Group	Value
Maternal characteristic		
Age (years) *^a^*		
	18∼	110 (18.90)
	25∼	290 (49.80)
	30∼	182 (31.30)
height (cm) *^b^*		159.68 ± 5.10
Prepregnancy BMI group *^a^*		
	Underweight	35 (6.00)
	Normal weight	338 (58.10)
	Overweigh	133 (22.90)
	Obesity	76 (13.10)
Family’s monthly economic income *^a^*		
	2000 yuan or less	107 (18.40)
	2000 yuan to 3000 yuan	244 (41.90)
	3000 yuan and above	231 (39.70)
Maternal Educational level *^a^*		
	Junior high school and below	374 (64.30)
	High School/Vocational High School/Secondary Technical School	88 (15.10)
	College degree or above	120 (20.60)
Intensity of physical labor *^a^*		
	light physical labor	337 (57.9)
	Moderate physical labor	46 (7.90)
	heavy physical labour	199 (34.20)
Maternity history *^a^*		
	primiparity	162 (27.87)
	multiparity	420 (72.20)
Secondhand smoke during pregnancy *^a^*		
	yes	
	no	
Weight gain during pregnancy (kg) *^b^*		15.67 ± 5.43
Gestational diabetes mellitus *^a^*		
	yes	11 (1.9)
	no	483 (83.0)
	unclear	88 (15.1)
Pregnancy-induced hypertension syndrome *^a^*		
	yes	86 (14.8)
	no	352 (60.5)
	unclear	144 (24.7)
Nutritional supplement use during pregnancy *^a^*		
	yes	367 (63.1)
	no	215 (36.9)
Smoking history during the six months preceding pregnancy in pregnant women *^a^*		
	yes	4 (0.7)
	no	578 (99.3)
Alcohol consumption during the six months preceding pregnancy in pregnant women *^a^*		
	yes	5 (0.9)
	no	577 (99.1)
Father’s characteristics		
Father’s Body Mass Index (BMI) *^a^*		
	Underweight	16 (2.7)
	Normal weight	201 (34.5)
	Overweigh	254 (43.6)
	Obesity	111 (19.1)
Father’s education level *^a^*		
	Junior high school and below	387 (66.5)
	High School/Vocational High School/Secondary Technical School	82 (14.1)
	College degree or above	113 (19.4)
Preschool Children Characteristics		
sex *^a^*		
	male	293 (50.30)
	female	289 (49.70)
gestational weeks *^a^*	<39 weeks	133 (22.90)
	≥39 weeks	449 (77.10)
birth height (cm) *^b^*		50.06 ± 0.99
birth weight (g) *^b^*		3396.54 ± 449.28
feeding patterns		
	Exclusive breastfeeding	432 (74.20)
	Formula feeding	150 (25.80)
Follow-up of the past month for children aged 3 to 6 years		
Number of children		582
Age (years) *^b^*		4.43 ± 1.11
Height (cm) *^b^*		105.95 ± 6.92
Weight (kg) *^b^*		17.48 ± 3.03
Body mass index BMI (kg/m^2^) *^b^*		15.49 ± 1.53
Body Fat Percentage PBF (%) *^b^*		16.703 ± 5.639
Fat Mass Index FMI (kg/m^2^) *^b^*		2.660 ± 1.181
Lean mass index FFMI (kg/m^2^) *^b^*		12.858 ± 0.738
Skeletal Muscle Mass Index SMI (kg/m^2^) *^b^*		4.106 ± 0.702
Daily intake of grains (g/d) *^b^*		73.32 ± 30.87
Daily intake of tubers (g/d) *^b^*		21.18 ± 17.14
Daily milk intake (g/d) *^b^*		71.15 ± 53.79
Daily intake of legumes (g/d) *^b^*		1.83 ± 2.24
Daily meat intake (g/d) *^b^*		43.68 ± 22.51
Daily egg intake (g/d) *^b^*		25.14 ± 12.94
Daily fruit intake (g/d) *^b^*		86.10 ± 43.06
Daily intake of dark-colored vegetables (g/d) *^b^*		19.22 ± 18.02
Daily intake of light-colored vegetables (g/d) *^b^*		66.24 ± 37.23
Daily intake of bacteria and algae (g/d) *^b^*		0.87 ± 1.37
Daily nut intake (g/d) *^b^*		0.94 ± 1.88
Daily snack intake (g/d) *^b^*		21.36 ± 17.14
Daily intake of beverages (ml/d) *^b^*		2.73 ± 6.49
Daily water intake (ml) *^c^*		1115.00 (882.00, 1290.00)
Currently taking nutritional supplements *^a^*		
	yes	273 (46.90)
	no	304 (52.20)
	Missing	5 (0.90)
Daily duration of indoor activities (h/d) *^b^*		2.91 ± 1.20
Daily outdoor activity duration (h/d) *^b^*		2.65 ± 0.79
Daily static activity duration (h/d) *^b^*		2.27 ± 0.85
Daily screen time (h/d) *^b^*		1.41 ± 0.74
Daily sleep duration (h/d) *^b^*		11.63 ± 1.07
Inquire whether diarrhea occurred within the preceding month *^a^*		
	yes	5 (0.90)
	no	577 (99.10)
Inquire whether the patient had a cold or fever one month prior to the investigation *^a^*		
	yes	154 (26.50)
	no	428 (73.50)
Check whether pneumonia was contracted within one month prior to the investigation *^a^*		
	yes	18 (3.10)
	no	564 (96.90)

Values are absolute numbers; *^a^*, number (percentage); *^b^*, mean ± standard deviation; *^c^*, median [Q1, Q3].

**Table 2:** Distribution of the rotated factor loading matrix.

Food Group	Dietary Pattern				
	Pattern 1	Pattern 2	Pattern 3	Pattern 4	Pattern 5
cereal		0.737			
Tubers		0.723			
Bean and pulses	0.432			0.499	
Dark vegetable				0.503	
Light vegetable	0.307	0.685			
Mushroom and algae	0.393	0.308			
Fruit		0.688		−0.319	
Milk			0.628		
Eggs	0.660				
Red meat	0.398		0.355		
Poultry meat	0.688				
Meat products			0.818		
Fish, shrimp and other aquatic products	0.744				
Cured vegetable					0.922
Bread, biscuits, chocolate and other snacks	0.526				
Nuts	0.301	0.371		−0.595	

**Table 3:** Association between maternal dietary patterns before pregnancy and body composition in offspring.

Dietary Pattern	PBF		FMI		FFMI		SMI	
	Crude	Multivariable	Crude	Multivariable	Crude	Multivariable	Crude	Multivariable
Pattern1								
Q1 low	Reference	Reference	Reference	Reference	Reference	Reference	Reference	Reference
Q2	−0.079 (−0.308, 0.151)	−0.038 (−0.263, 0.187)	0.039 (−0.192, 0.269)	−0.030 (−0.254, 0.194)	−0.041 (−0.271, 0.190)	−0.062 (−0.276, 0.151)	0.018 (−0.212, 0.249)	−0.027 (−0.249, 0.194)
Q3	0.163 (−0.067, 0.393)	0.131 (−0.104, 0.367)	0.103 (−0.128,0.333)	0.130 (−0.105, 0.365)	−0.042 (−0.273, 0.189)	−0.111 (−0.335, 0.113)	0.011 (−0.220, 0.242)	−0.022 (−0.254, 0.210)
Q4 high	0.175 (−0.055, 0.404)	0.041 (−0.210, 0.292)	0.033 (−0.198, 0.263)	0.015 (−0.235, 0.266)	−0.154 (−0.385, 0.076)	−0.149 (−0.387, 0.090)	−0.049 (−0.280, 0.180)	−0.044 (−0.292, 0.203)
*p* value, per SD	0.01 *	0.177	0.013 *	0.199	0.927	0.697	0.446	0.258
Pattern 2								
Q1 low	Reference	Reference	Reference	Reference	Reference	Reference	Reference	Reference
Q2	0.114 (−0.116, 0.345)	0.143 (−0.088, 0.375)	0.039 (−0.192, 0.269)	0.061 (−0.170, 0.291)	−0.305 (−0.534, −0.075) *	−0.366 (−0.586, −0.146) *	−0.340 (−0.569, −0.111) *	−0.335 (−0.563, −0.108) *
Q3	0.154 (−0.076, 0.384)	0.127 (−0.115, 0.368)	0.103 (−0.128, 0.333)	0.081 (−0.160, 0.321)	−0.201 (−0.430, 0.028)	−0.273 (−0.502, −0.043) *	−0.288 (−0.516, −0.059) *	−0.244 (−0.482, −0.006) *
Q4 high	0.065 (−0.166, 0.295)	0.101 (−0.157, 0.358)	0.033 (−0.198, 0.263)	0.080 (−0.177, 0.337)	−0.155 (−0.385, 0.074)	−0.265 (−0.510, −0.020) *	−0.267 (−0.495, −0.038) *	−0.256 (−0.510, −0.002) *
*p* value, per SD	0.870	0.975	0.750	0.855	0.244	0.059	0.036	0.012 *
Pattern 3								
Q1 low	Reference	Reference	Reference	Reference	Reference	Reference	Reference	Reference
Q2	−0.176 (−0.405, 0.053)	−0.127 (−0.367, 0.113)	−0.140 (−0.368, 0.089)	−0.011 (−0.350, 0.128)	−0.073 (−0.303, 0.158)	−0.156 (−0.384, 0.072)	−0.128 (−0.358, 0.102)	−0.247 (−0.483, −0.011)
Q3	0.051 (−0.178, 0.280)	0.078 (−0.148, 0.305)	0.091 (−0.138, 0.320)	0.117 (−0.109, 0.343)	0.043 (−0.187, 0.274)	0.049 (−0.166, 0.264)	−0.063 (−0.293, 0.167)	−0.059 (−0.282, 0.164)
Q4 high	0.202 (−0.027, 0.431)	0.215 (−0.012, 0.442)	0.233 (0.004, 0.462) *	0.262 (0.036, 0.488) *	0.058 (−0.173, 0.289)	0.137 (−0.079, 0.352)	0.109 (−0.121, 0.339)	0.169 (−0.054, 0.393)
*p* value, per SD	<0.01 *	<0.01*	<0.01 *	<0.01 *	0.920	0.539	0.419	0.145
Pattern 4								
Q1 low	Reference	Reference	Reference	Reference	Reference	Reference	Reference	Reference
Q2	0.058 (−0.173, 0.288)	0.182 (−0.050, 0.415)	0.038 (−0.193, 0.268)	0.137 (−0.095, 0.368)	−0.066 (−0.296, 0.164)	−0.186 (−0.407, 0.034)	−0.033 (−0.262, 0.195)	−0.094 (−0.323, 0.134)
Q3	0.116 (−0.114, 0.346)	0.234 (0.007, 0.461) *	0.085 (−0.145, 0.315)	0.201 (−0.025, 0.427)	−0.190 (−0.420, 0.039)	−0.206 (−0.421, 0.009)	−0.218 (−0.447, 0.010)	−0.188 (−0.412, 0.035)
Q4 high	−0.003 (−0.233, 0.228)	0.043 (−0.186, 0.272)	−0.072 (−0.302, 0.159)	−0.029 (−0.257, 0.199)	−0.174 (−0.405, 0.056)	−0.206 (−0.423, 0.011)	−0.342 (−0.570, −0.113)	−0.338 (−0.564, −0.113) *
*p* value, per SD	0.421	0.633	0.102	0.173	0.014 *	0.011 *	0.001 *	0.001 *
Pattern 5								
Q1 low	Reference	Reference	Reference	Reference	Reference	Reference	Reference	Reference
Q2	−0.214 (−0.444, 0.017)	−0.096 (−0.336, 0.144)	−0.177 (−0.407, 0.054)	−0.031 (−0.270, 0.208)	0.099 (−0.131, 0.330)	0.224 (−0.004, 0.452)	−0.056 (−0.286, 0.174)	0.116 (−0.120, 0.352)
Q3	−0.157 (−0.388, 0.073)	−0.035 (−0.271, 0.202)	−0.086 (−0.317, 0.144)	0.060 (−0.175, 0.295)	0.141 (−0.089, 0.372)	0.257 (0.033, 0.481) *	0.032 (−0.198, 0.261)	0.197 (−0.036, 0429)
Q4 high	−0.109 (−0.339, 0.122)	−0.074 (−0.309, 0.160)	−0.097 (−0.328, 0.133)	−0.035 (−0.268, 0.199)	0.071 (−0.160, 0.302)	0.177 (−0.045, 0.400)	−0.224 (−0.454, 0.006)	−0.042 (−0.273, 0.188)
*p* value, per SD	0.246	0.314	0.260	0.309	0.392	0.344	0.215	0.472

Note: Pattern 1: Anima-based food pattern; Pattern 2: Grain-Vegetable-fruit pattern; Pattern 3: Processed Meat-Dairy Pattern; Pattern 4: Vegetable-Legume Pattern; Pattern 5: Pickled Vegetable pattern. Calculate the dietary pattern scores and group them by quartiles, where Q1 and Q4 represent the low-score and high-score groups for each dietary pattern, respectively; the interval values for each group are indicated. * indicates *p* < 0.05. (The “crude model” refers to the unadjusted model; the “multivariable model” refers to the model adjusted for confounding factors, including the other dietary patterns, offspring sex, birth weight, birth length, maternal parity, maternal age at delivery, education level, physical activity, pre-pregnancy BMI, family monthly income, feeding mode, paternal BMI, secondhand smoke exposure during pregnancy, use of nutritional supplements during pregnancy, gestational diabetes mellitus, gestational hypertensive disorders, and gestational weight gain).

## Data Availability

The data supporting the findings of this study are available from the corresponding author, Changqing Liu, upon reasonable request.

## Supplementary Materials

The following supporting information can be downloaded at: https://www.mdpi.com/article/10.3390/nu18172895/s1, Figure S1: Scree Plot; Table S1: Distribution of the rotated factor loading matrix; Table S2: Test of the main effects and interaction between maternal pre-pregnancy dietary patterns and body composition in preschool-aged children; Table S3: Benjamini-Hochberg FDR Correction for Multiple Comparisons *; Figure S2: Regression diagnostic plots for multivariable linear models across four body composition outcomes.
