# Supplementary material for: Associations Between Maternal Pre-Pregnancy Dietary Patterns and Body Composition in Preschool Children: A Prospective Cohort Study
Source: Nutrients. 2026 Sep 3;18(17):2895. doi: 10.3390/nu18172895 (PMC13567508; doi:10.3390/nu18172895)
Supplement: Supplementary file 1 [file nutrients-18-02895-s001.zip › nutrients-4486177-supplementary.pdf]

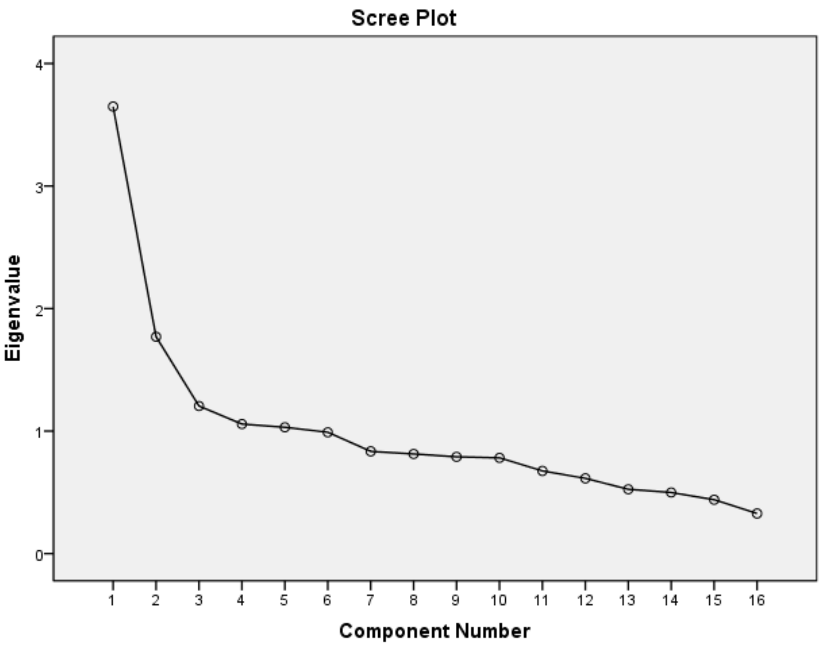

Figure S1 Scree Plot

Table S1 Distribution of the rotated factor loading matrix

	Dietary pattern				
	1	2	3	4	5
cereal	-0.008	0.737	0.097	0.205	0.024
Tubers	0.010	0.723	-0.045	0.070	-0.096
Bean and pulses	0.432	0.205	-0.025	0.499	-0.108
Eggs	0.660	-0.075	0.124	-0.072	-0.118
Milk	0.149	0.157	0.628	-0.273	-0.171
Fruit	0.063	0.688	0.119	-0.319	0.068
Dark vegetable	-0.062	0.243	-0.030	0.503	0.105
Light vegetable	0.307	0.685	0.283	0.194	0.135
Cured vegetable	0.000	-0.005	0.007	-0.008	0.922
Mushrooms and algae	0.393	0.308	0.191	-0.051	0.299
Red meat	0.398	0.128	0.355	0.018	0.087
Poultry meat	0.688	0.001	0.231	0.062	0.028
Meat products	0.118	0.034	0.818	0.144	0.125
Fish,shrimp and other aquatic products	0.744	0.089	-0.113	-0.044	0.067
Bread, biscuits, chocolate and other snacks	0.526	0.163	0.266	-0.285	0.057

Nuts	0.301	0.371	-0.067	-0.595	0.160

Table S2 Test of the main effects and interaction between maternal pre-pregnancy dietary patterns and body composition in preschool-aged children

variable	Dietary pattern	Main effect β (95% CI)	Main effect P	Gender interaction P	Preconception BMI interaction P
**PBF**	pattern1	0.059(-0.027,0.144)	0.177	0.173	0.251
	pattern2	0.001(-0.081,0.083)	0.975	0.890	0.370
	pattern3	0.147(0.069,0.225)	<0.001*	0.497	0.509
	pattern4	-0.019(-0.098,0.060)	0.633	0.579	0.184
	pattern5	0.041(-0.039,0.122)	0.314	0.857	0.513
**FMI**	pattern1	0.056(-0.029,0.141)	0.199	0.317	0.232
	pattern2	-0.008(-0.089,0.074)	0.855	0.752	0.232
	pattern3	0.153(0.076,0.230)	<0.001*	0.550	0.475
	pattern4	-0.054(-0.133,0.024)	0.173	0.246	0.288
	pattern5	0.041(-0.039,0.121)	0.309	0.890	0.394
**FFMI**	pattern1	0.016(-0.066,0.098)	0.697	0.048*	0.534
	pattern2	-0.076(-0.154,0.003)	0.059	0.495	0.176
	pattern3	0.023(-0.051,0.098)	0.539	0.577	0.200
	pattern4	-0.098(-0.174,-0.023)	0.011*	0.257	0.736
	pattern5	0.037(-0.040,0.114)	0.344	0.350	0.116
**SMI**	pattern1	0.049(-0.036,0.134)	0.258	0.013*	0.932
	pattern2	-0.104(-0.185,-0.023)	0.012*	0.234	0.051
	pattern3	0.058(-0.020,0.135)	0.145	0.786	0.033*
	pattern4	-0.128(-0.207,-0.050)	0.001*	0.185	0.210
	pattern5	-0.029(-0.109,-0.051)	0.472	0.548	0.420

Note: Table S2 presents overall main-effect estimates and effect-modification tests. The main-effect β (95% CI) and P-values were derived from multivariable-adjusted models without interaction terms, corresponding to the per-SD continuous associations shown in Table 3. The gender interaction P-values were obtained from separate models additionally including the interaction term of dietary pattern × offspring sex. The pre-pregnancy BMI interaction P-values were obtained from another set of separate models additionally including dietary pattern × pre-pregnancy BMI. * indicates P < 0.05. All models were adjusted for other dietary patterns, offspring sex (where applicable), birth weight, birth length, maternal parity, maternal age at delivery, education level, physical activity, pre-pregnancy BMI, monthly household income, feeding mode, paternal BMI, second-hand smoke exposure during pregnancy, use of nutritional supplements during pregnancy, gestational diabetes mellitus, pregnancy-induced hypertension, and gestational weight gain.

Multiple testing was addressed by Benjamini–Hochberg FDR correction, applied separately to three families of 20 tests (main effects, sex interactions, BMI interactions). Three main effects were significant after FDR adjustment (FDR < 0.05), but no interaction test yielded an adjusted P below 0.05, even though some had nominal P values of 0.048, 0.013 (sex) and 0.033 (BMI). We therefore caution that these interaction results are only

suggestive and warrant further investigation; they should not be interpreted as confirmatory.

Table S3. Benjamini-Hochberg FDR Correction for Multiple Comparisons*

FDR-adjusted P < 0.05

Outcome	Dietary Pattern	Main Effect Nominal P	Main Effect FDR-adj P	Sex Interaction Nominal P	Sex Interaction FDR-adj P	BMI Interaction Nominal P	BMI Interaction FDR-adj P
**PBF**	pattern1	0.177	0.393	0.173	0.734	0.251	0.502
	pattern2	0.975	0.975	0.890	0.890	0.370	0.593
	pattern3	<0.001*	0.005*	0.497	0.772	0.509	0.593
	pattern4	0.633	0.745	0.579	0.772	0.184	0.502
	pattern5	0.314	0.483	0.857	0.890	0.513	0.593
**FMI**	pattern1	0.199	0.398	0.317	0.772	0.232	0.502
	pattern2	0.855	0.900	0.752	0.890	0.232	0.502
	pattern3	<0.001*	0.005*	0.550	0.772	0.475	0.593
	pattern4	0.173	0.393	0.246	0.734	0.288	0.524
	pattern5	0.309	0.483	0.890	0.890	0.394	0.593
**FFMI**	pattern1	0.697	0.774	0.048	0.480	0.534	0.593
	pattern2	0.059	0.197	0.495	0.772	0.176	0.502
	pattern3	0.539	0.674	0.577	0.772	0.200	0.502
	pattern4	0.011*	0.048*	0.257	0.734	0.736	0.775
	pattern5	0.344	0.491	0.350	0.772	0.116	0.502
**SMI**	pattern1	0.258	0.469	0.013	0.260	0.932	0.932
	pattern2	0.012*	0.048*	0.234	0.734	0.051	0.502
	pattern3	0.145	0.393	0.786	0.890	0.033	0.502
	pattern4	0.001*	0.007*	0.185	0.734	0.210	0.502

Outcome	Dietary Pattern	Main Effect Nominal P	Main Effect FDR-adj P	Sex Interaction Nominal P	Sex Interaction FDR-adj P	BMI Interaction Nominal P	BMI Interaction FDR-adj P
	pattern5	0.472	0.629	0.548	0.772	0.420	0.593

Table S3. Benjamini-Hochberg FDR Correction for Multiple Comparisons*.FDR-adjusted P < 0.05.Note: Three independent hypothesis families were defined: (i) 20 main-effect tests (5 dietary patterns × 4 body composition outcomes); (ii) 20 sex-interaction tests; and (iii) 20 BMI-interaction tests. The Benjamini-Hochberg false discovery rate (FDR) correction was applied separately within each family.

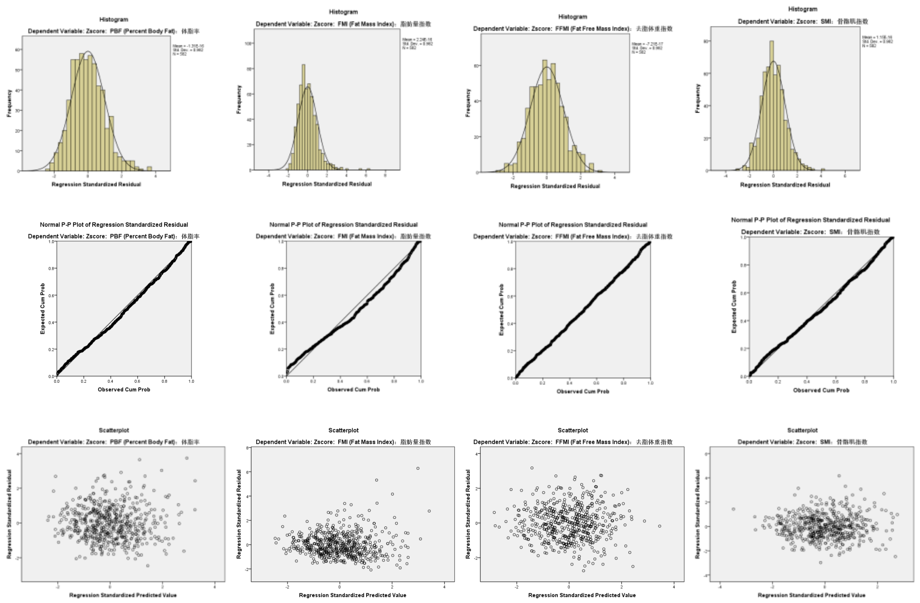

Figure S2. Regression diagnostic plots for multivariable linear models across four body composition outcomes. For each outcome (PBF, FMI, SMI, and FFMI), three diagnostic plots are presented: (left column) histogram of standardized residuals with normal curve overlay; (middle column) normal P–P plot of standardized residuals; (right column) scatterplot of standardized residuals against standardized predicted values. All plots indicate adequate satisfaction of linear regression assumptions, with no serious multicollinearity (VIF < 2.0), no substantial departure from residual normality, no discernible

heteroscedasticity or non-linearity, and no influential observations (Cook's distance < 1.0).
